# 219. A Randomized, Double-Masked, Placebo-Controlled Phase IIB Trial of Azithromycin and Trimethoprim-Sulfamethoxazole as Bacterial STI Prophylaxis in Pregnant Women with HIV

**DOI:** 10.1093/ofid/ofac492.297

**Published:** 2022-12-15

**Authors:** Jodie Dionne, Barbara Van Der Pol, Dustin Long, Pius Tih, Rahel Mbah, Edward Ngah, Seraphine Pekwarake, Mirabelle Kifem, Anthony Fondzeyuf, Katia J Bruxvoort, Alan Tita, Jeanne Marrazzo

**Affiliations:** University of Alabama at Birmingham, Birmingham, Alabama; UAB, Birmingham, Alabama; UAB, Birmingham, Alabama; CBCHS, Bamenda, Nord-Ouest, Cameroon; CBCHS, Bamenda, Nord-Ouest, Cameroon; CBCHS, Bamenda, Nord-Ouest, Cameroon; CBCHS, Bamenda, Nord-Ouest, Cameroon; CBCHS, Bamenda, Nord-Ouest, Cameroon; CBCHS, Bamenda, Nord-Ouest, Cameroon; University of Alabama at Birmingham, Birmingham, Alabama; UAB, Birmingham, Alabama; UAB, Birmingham, Alabama

## Abstract

**Background:**

This phase IIB randomized clinical trial was designed to test the efficacy of a novel regimen to prevent bacterial sexually transmitted infections and malaria in pregnant women with HIV in Cameroon, where HIV prevalence in pregnancy is 5.7%. Here we present the analysis of STI rates.

**Methods:**

Pregnant women in prenatal care with confirmed HIV, gestational age < 28 weeks and singleton pregnancies were randomized to monthly azithromycin (AZ) 1 gram daily for 3 days and daily trimethoprim-sulfamethoxazole (TMPS) or the standard regimen of daily TMPS with monthly placebo AZ.

The main outcome of interest was the proportion of women with a composite STI measure: chlamydia, gonorrhea and/or incident syphilis at delivery. Nucleic acid amplification testing (NAAT) for CT/NG was performed on provider-collected vaginal swabs. Incident syphilis was defined serologically as a newly positive treponemal test or 4 fold increase in RPR/VDRL titer since baseline. The proportion was compared by relative risk with 95% confidence intervals and a significant p value set at < 0.05.

**Results:**

A total of 308 women were enrolled at three hospital facilities between March 2018 and August 2020. In all, 155 women were randomized to the AZ/TMPS arm and 153 women to the TMPS arm. A total of 260 women (84%) had delivery samples collected. Both groups were similar with median age 32 years, maternal education (71% secondary school or university), HIV diagnosis 3 years prior, and 94% reported excellent adherence to antiretroviral therapy (ART). Median CD4 count was 473 cells/mm3 (IQR 326-663). At baseline, prevalence of chlamydia was 1.4%, gonorrhea 1.0% and syphilis 1.9%. There was no difference in the proportion of women with the composite STI measure (3.2% in the AZ/TMPS arm and 3.3% in the TMPS arm; RR 0.78 (95% CI 0.21 – 2.84); p=0.70). Adverse birth outcomes were lower in the AZ/TMPS arm, but not significantly (preterm delivery 5% vs 10.3% [p=0.1], low birthweight 2.8% vs 5.1% [p=0.34], composite adverse birth outcome 8.4% vs 13.1% [p=0.19].

**Table 1:**
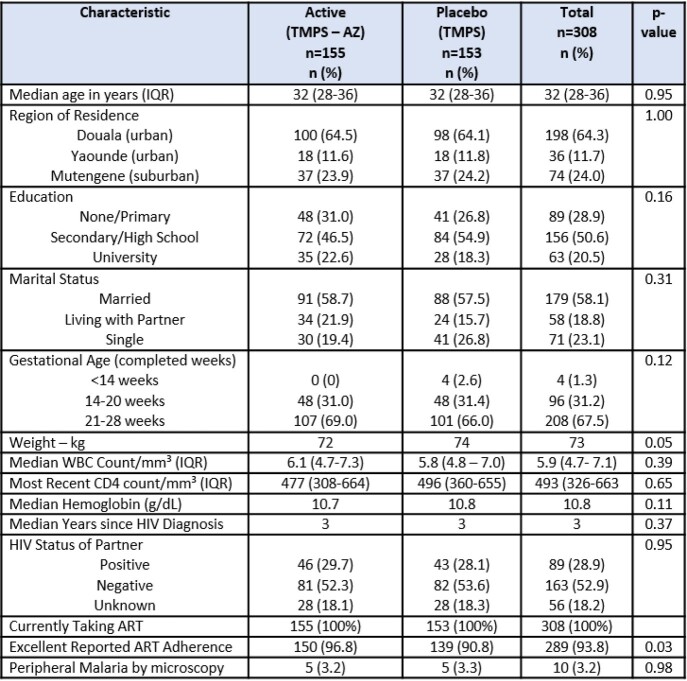
Baseline Characteristics of Study Participants (n=308)

**Table 2:**
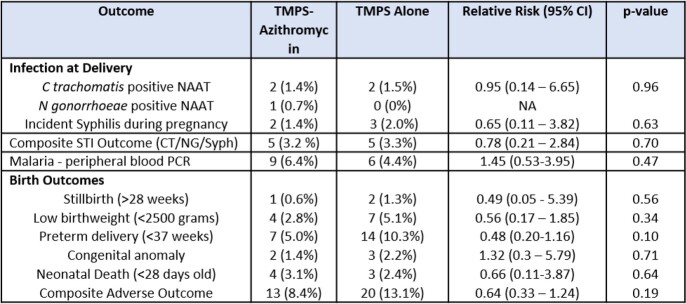
Efficacy Outcomes at Delivery by Arm - Intention to Treat Analysis (n= 278)

**Conclusion:**

The addition of monthly azithromycin to standard daily TMPS prophylaxis in pregnant women living with HIV in Cameroon did not reduce the rate of bacterial STI at delivery. Women reported excellent ART adherence and rates of STI, malaria, and adverse birth outcome were low.

**Disclosures:**

**Katia J. Bruxvoort, PhD, MPH**, Dynavax: Grant/Research Support|Gilead: Grant/Research Support|Glaxosmithkline: Grant/Research Support|Moderna: Grant/Research Support|Pfizer: Grant/Research Support|Seqirus: Grant/Research Support.

